# Effect of assisted walking-movement in patients with genetic and acquired neuromuscular disorders with the motorised Innowalk device: an international case study meta-analysis

**DOI:** 10.7717/peerj.7098

**Published:** 2019-06-18

**Authors:** Caroline Schmidt-Lucke, Jana Käferle, Britt-Marie Rydh Berner, Lotta Ahlborg, Hege Marie Hansen, Ulrika Skjellvik Tollefsen, Tonje Thon, Rikke Damkjær Moen, Ana Pekanovic, Åsa B. Tornberg, Katarina Lauruschkus

**Affiliations:** 1 Charité University Berlin, Berlin, Germany; 2 Medico-academic Consultings, Berlin, Germany; 3 Donau-Universität Krems, Krems, Austria; 4 Department of Rehabilitation Medicine Stockholm, Danderyd University Hospital, Stockholm, Sweden; 5 University of Bergen, Bergen, Norway; 6 Municipality of Asker, Asker, Norway; 7 Municipality of Porsgrunn, Posgrunn, Norway; 8 Made for Movement, Skien, Norway; 9 Department of Health Sciences, Faculty of Medicine, Lund University, Lund, Sweden

**Keywords:** Rehabilitation, Neuromuscular disorder, Physical excersise, Medical device

## Abstract

People with physical disabilities (PD) suffer from consequences due to lack of physical activity and consequently, are at increased risk of chronic diseases. We aimed to evaluate the ability of a motorised assistive device for dynamic standing with weight-bearing in addition to standard state-of-the-art therapy to improve clinical outcome in a meta-analysis of available studies. A total of 11 studies were identified from different European countries analysing the effect of the dynamic device Innowalk. Raw data of nine studies were pooled including a total of 31 patients observed between 2009 and 2017. Standardised questionnaires and physical outcomes were examined in this exploratory meta-analysis. We recorded patients’ characteristics, duration, intensity, and location of usage as well as general clinical outcomes and improvement of passive range of motion (PROM). The analysed population consisted in 90% cases of patients younger than 18 years of age. Patients were severely disabled individuals (aged 8 (6–10) years; 58% male; 67% non-ambulatory, 86% cerebral palsy). A total of 94% used the Innowalk in a home-based or day-care setting. For nearly all individuals (94%), improvements were recorded for: walking or weight-bearing transfer (*n* = 13), control/strength of the trunk or head (*n* = 6), joint mobility (*n* = 14), sleep (*n* = 4 out of 6/67%), or muscle strength (*n* = 17), vital functions (*n* = 16), bowel function (*n* = 10), attention/orientation (*n* = 2). PROM of the hip (flexion, abduction, and adduction) significantly (*p* < 0.001 for multiple comparisons) increased after 1 month (*p* < 0.05 flexion, adduction) and further after 5 months (*p* < 0.05 each) in contrast (*p* < 0.05 each) to a control group with state-of-the-art therapy. Similarly, PROM showed a trend towards improvement in dorsal extension of the ankle (*p* = 0.07). In summary, this is the first report of a novel device with additional benefit to standard therapy for severe PD. These intriguing results warrant the planned prospective randomised controlled trial to prove the concept and mechanism of action of this device.

## Introduction

Physical disabilities (PD) with permanent motor impairments, due to non-progressive brain disorders, that is, cerebral palsy, are characterised by their complex symptoms, diverse underlying aetiology, and consequent disabling disease progression. These heterogeneous entities often result in pathologic muscle activity, imbalance between agonists and antagonists with consequent contractures and deformities (Deutsche Gesellschaft für Neurologie, 2012; Rosenbaum et al., 2002). Development is characterised by perpetual muscle mass and strength loss, as well as increase in discomfort, spasticity, reduced joint mobility, and overall decline in quality of life (Rosenbaum et al., 2002). Multidisciplinary treatment aims to improve quality of life of patients and caregivers as well as to prevent secondary damages through pathologic movement patterns, growth and positioning problems. In addition to targeting sensor monitoring and social cognitive development, spasticity requires reduction of pathologic high muscle tone. Muscle weakness or disturbances of coordination require orthopaedic devices; structural changes require improvement of muscle balance and restoration of physiological level ratio (Thilmann, Fellows & Garms, 1991; Armand et al., 2005; Stotz, 2000).

Exercise and muscle activation have positive effects on muscle strength/function/tone, neuromusculoskeletal and movement-related functions, bladder and bowel function, endurance, flexibility, cardiorespiratory fitness, overall wellbeing, activities, and participation in people with PD (Maltais et al., 2014; Lauruschkus, Nordmark & Hallstrom, 2017; Novak et al., 2013; French, Fulkerson & Story, 2000). Cerebral palsy (PC) vs. other diagnoses, generally they have different requirements. Thus, according to recommendations, all individuals with PD should engage, to the extent they are able, in aerobic, anaerobic, and muscle-strengthening activities (Maltais et al., 2014; Ryan et al., 2017; Paleg, Smith & Glickman, 2013; Verschuren et al., 2016). Furthermore, the body of evidence suggests that the autonomic cardiac function is impaired in severely disabled patients with PD (Amichai & Katz-Leurer, 2014; Balemans et al., 2014; Park et al., 2002) correlating with their grade of gross motor function Gross Motor Function Classification System—Expanded & Revised (GMFCS-E&R) and a positive effect of activities on this system has been reported (Israeli-Mendlovic, Mendlovic & Katz-Leurer, 2014).

For these patients, multimodal treatment by a multidisciplinary team is costly, personnel-intensive and high-level scientific evidence is lacking. Especially for the hip, passive assisted motion of the legs with next-to-physiological gait-like motion in axial alignment may have a direct beneficial long-term effect to prevent hip deformities (Krebs, Strobl & Grill, 2008; Pountney & Green, 2006; Strobl, 2009) and thus, aid with weight-bearing transfer for daily activities.

Instability of the hip is a key feature for these patients. It occurs due to various interacting factors, such as changes of connective tissue, imbalance of the muscle tone between antagonistic muscle groups, reduced exposure to force of gravity, compensatory pathological movement patterns, or changes of the bones and joints adjacent to the hip (Strobl, 2009). Secondary changes include wringing of the pelvis, instability of the hip joints with progressive decentralisation as well as severe neuromuscular scoliosis (Strobl, 2009). In non-ambulatory individuals, progressive decentralisation of the hip joint develops leading to subluxation or luxation in 79% of non-ambulatory children with problems of weight-bearing, grooming pain, and significant reduction of quality of life (Strobl, 2009; Cornell, 1995; Heimkes, Stotz & Heid, 1992). In their 20-year follow-up (FU) study of a national quality register Uppföljningsprogram För Cerebral Pares (CPUP) with international participation, the authors (Hägglund et al., 2014) showed that hip dislocation could be prevented and the number of children who develop severe contractures and windswept deformity in scoliosis was reduced with adequate therapy.

Devices for dynamic standing, such as the EasyStand glider or the Innowalk device promote passive motion of the lower extremities during standing with weight-bearing. Whilst the EasyStand requires coordinated movement of the arms to move the legs, the Innowalk is the only device available that induces walking movements independent of functioning arm movements. In the Easy stand glider, no extension and flexion in the knee is possible, and the knees seem to be in an extended position, whereas, the Innowalk provides a more natural walking movement. The Innowalk has been specifically designed to be used at home or in institutions such as hospitals and exercise facilities. It is intended to be used by severely disabled (non-ambulatory) children and adults with neuromuscular disabilities related to diagnoses such as cerebral palsy, spina bifida, genetic disorders, traumatic, or hypoxic brain injury. Typically, these patients are unable to come into the upright position without adequate own movement capacity and ability to support their own weight. Furthermore, it is often impossible, for individuals with neuromuscular disorders, to reach the recommended minimum of 30 min for adults and 60 min for children, of moderate to vigorous physical activity minimum 5 days a week.

This assistive device makes it possible to reach the goal of regular daily physical activity in an upright weight-bearing position with the necessary and safe trunk support, while enabling passive movement of the legs. It has therefore been proposed to contribute to improvement of individual physical limitations (Lauruschkus, 2015). It was first prescribed in 2009 and since then it’s been available in more than 20 countries worldwide. Thus, therapy with the Innowalk as addition to state-of-the-art therapy such as physiotherapy may develop new ways to optimise performance of everyday tasks and participation.

We have gathered data from a network of Innowalk users (Käferle, 2014; Hansen, 2014; Thon, 2012; Moen, 2016; Tollefsen, 2015; Berner & Ahlborg, 2017; Tornes et al., 2012; Lauruschkus et al., 2017) from three European countries (Austria, Norway, and Sweden) in addition to standard state-of-the-art therapy such as physiotherapy, occupational therapy, use of orthotics and standers. So far, there has been no information gathered systematically regarding the prescription patterns, type of indications, age and gender distribution, symptoms or severity of disease or disability level, or the type of the Innowalk device used. This meta-analysis synthesizes the qualitative evidence about the medical benefit, impact on passive range of motion (PROM) of lower extremity joints, quality of life as well as define risks and undesired effects of a motorised assistive device for dynamic standing.

## Materials and Methods

### Search strategy

A comprehensive literature search was performed to identify all eligible studies. Electronic searches of PubMed, Google Scholar, Scopus, MEDLINE, Database of Reviews of Effectiveness, Cochrane Database of Systematic Reviews, CINAHL and ClinicalTrials.gov were performed in April 2018 using the key words ‘dynamic standing’, ‘EasyStand’, and/or ‘Innowalk’. Reference lists from the identified publications and presentations on congresses were used, the manufacturer of Innowalk was contacted and researchers knowledgeable about this intervention were consulted to identify other potential studies. No restrictions on the language of the publication were made. Determination of study status including prespecified quality criteria and data extraction based on pre-defined characteristics was performed by two independent reviewers (J.K. and A.P.). Following revision, decision to include data of a study into this meta-analysis was reached after consensus by three scientists (J.K., A.P., and C.S-L.).

The case studies have been conducted in Austria, Norway, and Sweden from 2009 to 2017. Written informed consents complying with individual national requirements were provided.

This analysis was done in accordance with the Declaration of Helsinki and was approved by the local Ethics Committee (Eth-15/2018) of Landesärztekammer Berlin (Berlin, Germany). The study has been registered in the PROSPERO database on 2nd October 2018 and has been assigned the number *CRD42018109076*. The process of data extraction from each study included was performed according to standard procedures (Darrah et al., 2008; The Cochrane Collaboration, 2011).

### Trials

There were two different devices that claimed to use dynamic standing, the EasyStand and the Innowalk.

Dynamic standing in the EasyStand requires addition of the so called gliders, in which the feet are fixed. To move the feet, the users have to move the glide handle actively with their arms to convey motion of the legs. This device is suitable for persons with spinal cord injuries rather than brain damage. Three trials using the EasyStand as passive standing frame only were found but none displayed features of dynamic standing (Paleg, Smith & Glickman, 2013; Paleg & Livingstone, 2015; Alizadeh-Meghrazi et al., 2012; Hadi et al., 2012; Totosy De Zepetnek et al., 2017). From direct contact with the manufacturer, no data on dynamic standing could be retrieved. Thus, these trials were not used for this evaluation.

**Figure 1:**
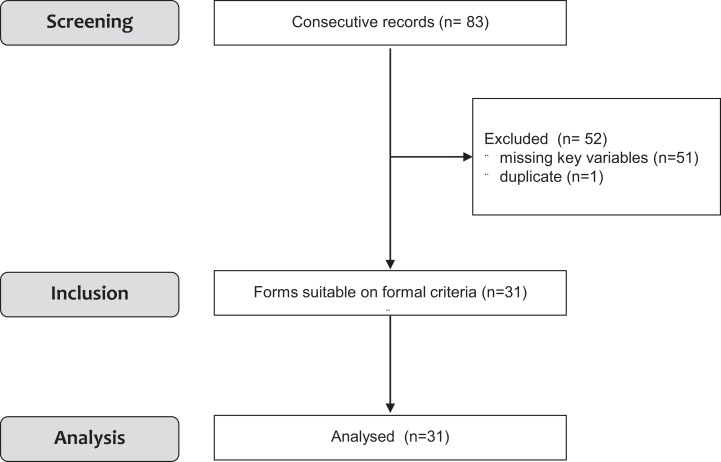
Flowchart of the study.

A total of 11 completed, non-randomised prospective studies, involving a total of 83 patients were identified that assessed the effects of dynamic standing with the Innowalk. Selection of patients for inclusion in this meta-analysis is presented in Fig. 1. The analysed studies were conducted from 2009 to 2017 (see Table 1) and were partially from unpublished, non-journal sources such as results from dissertations or abstracts from scientific meetings not yet published in peer-reviewed journals. Three additional ongoing studies (one in France and two in Sweden) could not be included in the analysis. In the studies used for this meta-analysis, not all patients’ data were complete and could be used. In one study (Tornes et al., 2012) only 4 out of 14 patients could be analysed. In the remaining 10 records, there were missing key variables that hampered analysis and data could not be retrieved. Of these patients, *n* = 4 are currently still using the Innowalk; *n* = 1 died due to complications due to the original diagnosis (brain injury); *n* = 1 stopped usage after improvement and reaching prespecified goals, no further data were available; in *n* = 2 lack of time/motivation of the parents to use the Innowalk was the reason for not further responding and further two patients were lost to FU. Raw data from 2 out of 11 studies could not be obtained in a high enough quality to be included (*n* = 30; Strobl et al., 2016; *n* = 11; Rosenstand & Fløistrup, 2009). One duplicate patient was identified in one case series and eliminated from the analysis. Thus, data from 31 individuals were analysed (Fig. 1). It was our aim to specifically detect changes in PROM of joints of the lower extremities. For this we searched whether all studies available had predefined and measured PROM as primary endpoint. Only two studies Käferle (2014) and Hansen (2014) had prespecified changes in PROM measurements using validated and standardised measurements. Thus, changes in PROM were derived from these two studies for this meta-analysis.

**Table 1:** Study characteristics—interventions and participants.

Author	Year	Level of evidence and conduct rating (Howick et al., 2011)	*n*	Indications	Gender	Age (in years)	Innowalk Use Frequency	Innowalk Use Duration	Goals set	Primary Outcome
Käferle (2014), Austria	2013	III	7 + 4^1^	Bilateral spastic CP	5 + 3 males^1^	6–10	5 days/weeks; 30 min	4 weeks	n. a.	Walking improvement, joint mobility improvement, muscle strength improvement; vital functions improvement
Hansen (2014), Norway	2014	V	2	Bilateral spastic CP; Bilateral spastic CP with micro encephalopathy	Two females	2–7	2–3 days/weeks	9–12 weeks	n. a.	Joint mobility improvement
Tornes et al. (2009), Norway	2009	IV	5	Spastic diplegic CP; spastic quadriplegic CP; acquired brain damage; dyskinetic CP; bilateral spastic CP	Three males	4–12	4–7 days/weeks; 30 min	4 weeks	n. a.	Walking improvement; joint mobility improvement; muscle strength improvement; vital function improvement
Moen (2016), Norway	2016	V	4	CP; Spastic quadriplegic CP	Four males	6–18	5–7 days/weeks; 30 min	n. a.	Movement improvement; walking improvement; muscle strength improvement; joint mobility improvement; vital functions improvement (bowels, circulation, sleep); prevention of deformities/contractures	Walking improvement; joint mobility improvement; muscle strength improvement; vital functions improvement
Tollefsen (2015), Norway	2015	V	1	Spinal muscular atrophy	One male	7	5 days/weeks; 45 min	4 weeks	Activity improvement; walking improvement; muscle strength improvement; prevention of deformities/contractures; transferring weight bear on legs	Vital functions improvement; muscle strength improvement
Tornes et al. (2012), Norway	2012	V	4	CP; Rett syndrome; bilateral spastic (Tollefsen, 2015) CP; acquired brain damage	Two males	2–11	3–4 days/weeks; 15–60 min	52 weeks	Joint mobility improvement; reduction of spasticity, prevention of deformities/contractures; improvement of vital functions (bowels, circulation, sleep); upper body strength; head control improvement	Walking improvement; joint mobility improvement; muscle strength improvement; vital functions improvement
Berner & Ahlborg (2017), Sweden	2017	V	2	Bilateral spastic CP	Two males	31–58	4–6 days/weeks; 36–60 min	n. a.	Activity, movement improvement; muscle strength improvement	Walking improvement; joint mobility improvement; muscle strength improvement; vital functions improvement
Lauruschkus et al. (2017), Sweden	2017	III	5	Bilateral spastic CP and bronchopulmonary dysplasia; dyskinetic CP; spastic bilateral CP; congenital muscular dystrophy	One male	8–11	2–5 days/weeks; 40–50 min	19 weeks	GAS; COPM	Vital functions improvement
Thon (2012), Norway	2012	V	1	Bilateral spastic CP	One female	13	6 days/weeks; 60 min	6 weeks	n. a.	Walking improvement; vital function improvement

**Note:**

1 Control subjects without usage of Innowalk.

### Study population

This meta-analysis includes the analysis of case studies of patients with various neuromuscular disabilities who used dynamic standing with the Innowalk as part of or in addition to their standard treatment, such as physiotherapy and behavioural therapy. This study was conceived as a post hoc re-analysis of raw data from nine described and partially published cohorts (Käferle, 2014; Hansen, 2014; Thon, 2012; Tornes et al., 2009, 2012; Moen, 2016; Tollefsen, 2015; Berner & Ahlborg, 2017; Lauruschkus et al., 2017). Investigators of each source study provided crude data on the basis of an agreed protocol and data scheme. The following data were recorded for all patients: age, gender, clinical indication, (GMFCS-E&R, for CP patients), size of Innowalk used, place of examination. Shared definitions of level of disability were used: The GMFCS-E&R (Palisano et al., 2008) and manual ability classification system (Eliasson et al., 2006). Acceptability of the device was recorded by the users and care-givers’ subjective impressions were recorded at the individual visits in three (Tornes et al., 2009; Moen, 2016; Lauruschkus et al., 2017) out of nine studies by using two different questionnaires. The standardised Innowalk protocol (Tornes et al., 2009; Moen, 2016) provided daily feedback on user’s experience and motivation, whereas goal attainment scale (GAS) (Lauruschkus et al., 2017) provided the effect of the dynamic standing with the Innowalk on an individual basis. One study (Lauruschkus et al., 2017) used the term ‘vital function’ to describe a combination of bowel function, sleep and attention.

### Follow-up and definition of the endpoint

In all source studies, FU was conducted by telephone contact, ambulatory, or home visits. Potentially undesired effects during or after usage of the device were verified by analysis of medical records, such as hospital charts, discharge letters, and personal communication with the caregivers. We analysed recorded improvements in PROM if they were pre-specified as an endpoint and if they had been documented in a standardised method (two studies; Hansen, 2014; Moen, 2016). Clinical improvement was measured after 4–52 weeks of usage (Käferle, 2014; Hansen, 2014; Thon, 2012; Tornes et al., 2009, 2012; Moen, 2016; Tollefsen, 2015; Berner & Ahlborg, 2017; Lauruschkus et al., 2017).

Occurrence of undesired effects during usage was inquired about at the beginning of each visit in the context of the assessment of the medical history in all case series. In two studies, a log book diary (Tollefsen, 2015; Lauruschkus et al., 2017) and in another a diary with pain scale were used to report undesired effects (Tornes et al., 2012). The remaining investigators documented undesired effects into the clinical patients’ records. As part of this meta-analysis, all investigators were prompted to provide their documentation of risk assessment and to review original records with regards to undesired effects as well as number of subjects withdrawn or pausing for longer (>1 week) time periods as result of undesired effects due to dynamic standing.

### The innowalk device

**Figure 2:**
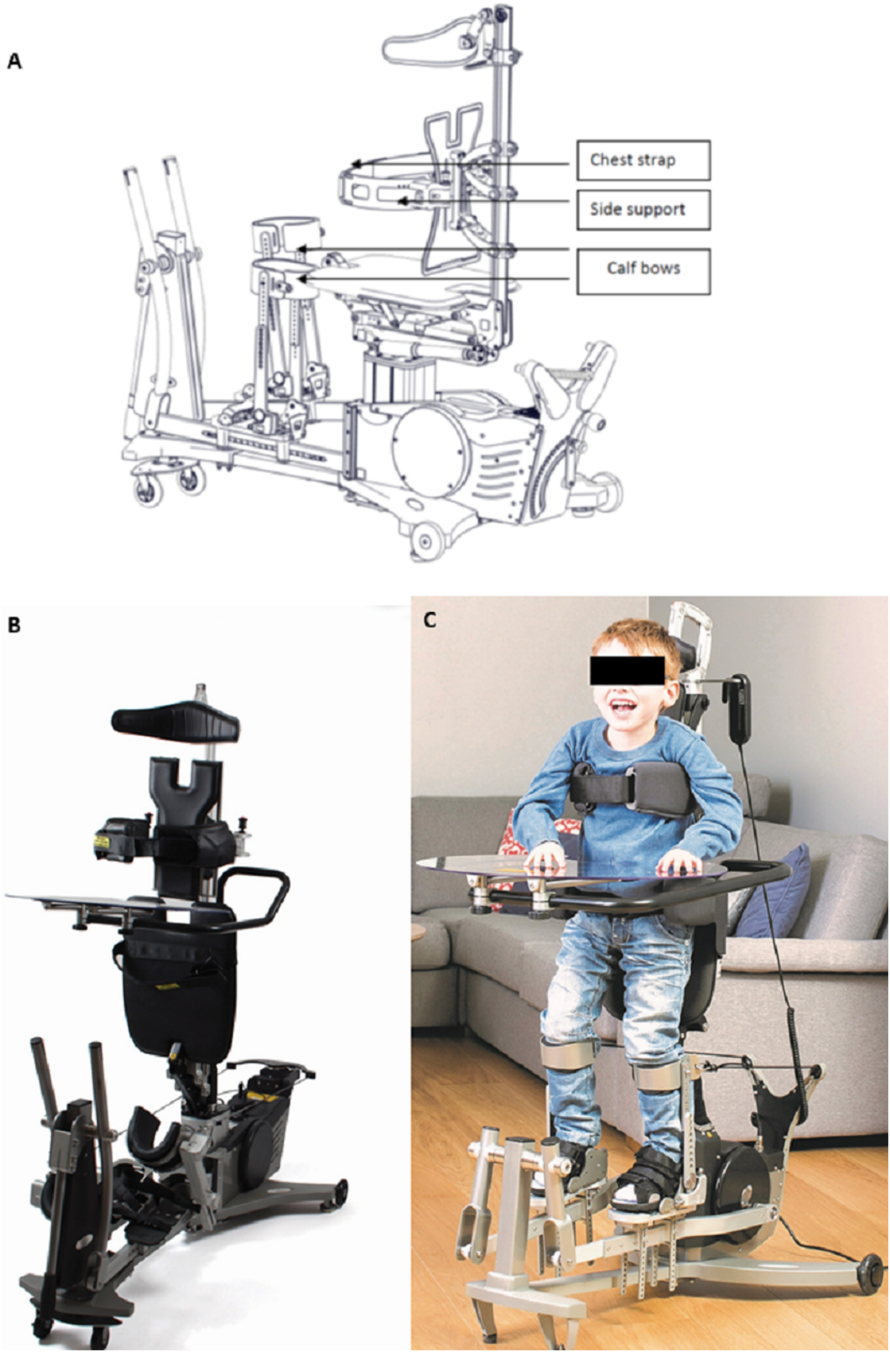
Dynamic standing device Innowalk. (A) Technical drawing of the Innowalk. (B) The Innowalk with table. (C) Child using the Innowalk in upright position with table. All images retrieved from Made for Movement Group AS; 2018 Copyright by Made for Movement Group AS.

The Innowalk (Made for Movement, Norway) is a motorised, multi-functional assistive device offering the possibility for assisted repetitive walking movements close to normal gait in an upright weight-bearing position (Fig. 2). This movement induces flexion and extension of the hip, knee, and ankle joints. Innowalk comprises a motor-driven gait orthosis for legs, a weight support system, neck support, shoulder straps, side-support with a belt, and a transport trolley. Before use, the device is connected to the power and a pre-check on the seat and upright function is performed. The device is in the sitting position when the user is transferred into the seat. The chest belt is secured first, following the guide-string attachment to the calf bow. Finally, the feet are secured with straps on the footplate. The user is then moved from a sitting to a standing position. When standing, the hip belt is secured and the movement of the legs can be turned on. Accessories can be added and attached if needed: table, anti-overstretch, shoulder straps, and handles for arm motion (Fig. 2). The handles for the arm movement can be used by those individuals who have some ability to grip the handles and hold on to them, and who have the PROM in the shoulder needed to perform the movement.

The device is individually adjusted to the size of the user in accordance to sitting height, sitting depth, leg length, and chest support height. Positioning of foot plates is regulated in accordance to PROM in angle, knees, and hips. Hip support is adjusted to the height of trochanter major in standing position. Head support is adjusted while standing. The angle of the seat (between sitting and full standing) is decided in relation to PROM or other deformities. The adaptation of above mentioned points is done at the first try out. Smaller adjustments are performed subsequently once or twice a year, depending on age and growth curve.

The speed of the pedal is started with around 10 rounds per minute (rpm) and increased depending on the patients’ individual tolerance to maximally 85 rpm via remote control. This can be adjusted within 4 s. The emergency stop can be used if the remote control is not working, in case of acute pain, discomfort, and epileptic seizure.

For security reasons, anti-spasticity control is included and can be adjusted according to the patients’ individual demands. Patients with uncontrolled epilepsy, skin abrasions, clinical relevant infectious disease, severe congenital disorders (i.e. congenital heart disease) should not use the Innowalk for safety reasons.

The responsible physiotherapist and the physician decide on the duration of usage that remains under continual evaluation by the person in the device and person assisting the user, due to tiredness, pain or other observational signs of discomfort.

We collected data on frequency of Innowalk use per week, length of the usage period (in weeks), and length of sessions (in minutes). Since this information has been given in different formats and raw data could not be extracted in a standardised manner, and furthermore, the actual use had not been documented in a standardised and comprehensible manner, these data could be presented for four out of nine studies (Käferle, 2014; Thon, 2012; Tornes et al., 2012; Lauruschkus et al., 2017).

### Risk of bias assessment

Two reviewers (A.P. and C.S.-L.) independently evaluated the five major domains of biases according to the Cochrane’s risk of bias tool (The Cochrane Collaboration, 2011):

– Selection bias: differences between experimental groups in terms of possible confounding prognostic factors.
– Performance bias: differences between groups in how and how long interventions were administered, presence of control group, randomisation or blinding.
– Detection bias: mode of determination of outcome by the use of validated and adequate questionnaires or blinding of the respective study’s investigator.
– Attrition bias: systematic group differences in the number of persons that quit or drop-outs or exclusion from analyses for any other reasons.
– Reporting bias: that is, the presence of differences between the reported (published) findings, and the initially planned and/or non-reported analyses, or whether at all endpoints had been pre-specified, including adverse events.
– Other bias: we additionally determined whether there were any other potential risk of biases, such as the absence of separate pre-tests to assess possible baseline differences in GMFCS-E&R between groups.

Two reviewers (A.P. and C.S.-L.) independently evaluated the included studies. Rating was performed manually by using the revised Cochrane risk-of-bias tool for randomised trials (RoB 2.0) (The Cochrane Collaboration, 2011). Risk of bias on each domain was scored using a set of predefined criteria. We specifically modified these criteria for the purpose of this review (Table S1). Individual items were scored ‘+’ for low risk of bias; ‘−’ for high risk of bias and ‘?’ for unclear risk of bias. Eventually, controlled trials were classified as low risk of bias (all items: ‘+’), moderate risk of bias (one or two items: ‘−’), or high risk of bias (>2 items: ‘−’). Trials were assigned an unclear risk of bias when four or more items were scored ‘?’. We next scored the corresponding overall ‘Level of Evidence’ in accordance to the table of Oxford’s Centre for Evidence Based Medicine (Howick et al., 2011).

### Statistical analysis

Continuous variables were tested for normal distribution with the Kolmogorov–Smirnov test. Comparisons between the two groups were analysed by *t*-test (two-sided) for normally distributed variables. Data are expressed as mean ± SD, unless otherwise stated. Non-normally distributed continuous variables (age, disability levels, duration and frequency of usage, and total hours of usage/month) were compared by the Mann–Whitney *U*-test for two groups or by the Kruskal–Wallis test with more than two subgroups with a post hoc analysis (Wilcoxon) for two dependent variables. To control for multiple comparisons in PROM of different joint angles and group comparisons (*n* = 10) the Bonferroni correction was applied to each *p*-value and the *uncorrected p*-values are reported in the text, tables, and graphs. Comparison of categorical variables was generated by the χ^2^ (Pearson, London, UK) test. SPSS versions 25.0 was used. Statistical significance was accepted at *p* < 0.005.

## Results

### Patient characteristics

#### Baseline

Characteristics of the studies are in Table 1. Nine studies, including one to seven patients, during the period from 2009 to 2017 were performed in Austria, Norway, and Sweden. Five studies had predefined goals (Moen, 2016; Tollefsen, 2015; Berner & Ahlborg, 2017; Tornes et al., 2012; Lauruschkus et al., 2017). The baseline demographics and clinical characteristics of all patients are summarised in Table 2. The study sample was representative of a severely disabled population with 90% being underage and 58% male, the majority presenting with spasticity. All but five patients had CP (86%). Other underlying or additional diagnoses were spinal muscular atrophy type 3, Rett syndrome, acquired brain injury, bronchopulmonary dysplasia, microcephaly, and congenital muscular dystrophy. GMFCS-E&R was classified at least as level IV in 78% of patients with CP. Only four patients were able to self-transfer or sit freely with none or minor difficulties. Out of 15 patients who provided information about pain, all but three pain-free patients reported mild (*n* = 8) to moderate (*n* = 4) pain. There was only incomplete information on weight or height, so we refrained from analysing and presenting this information.

**Table 2:** Baseline characteristics and demographic variables.

Characteristics	Frequencies or median (IQR)
Age	8 (6–10)
Age group 1: 2–11 years	26 (83.9%)
Age group 2: 12–17 years	2 (6.5%)
Age group 3: 18 and older	3 (9.7%)
Gender	
Male	18 (58.1%)
Indication	
CP	26 (86.2%)
With spasticity	15
With spasticity and paresis	1
And epilepsy	5
No specification	3
With musculoskeletal deformities and contractures	2
Other diagnosis^1^	5
GMFCS-E&R classification	
Level III	6 (19.4%)
Level IV	8 (25.8%)
Level V	13 (41.9%)
Not applicable^2^	Two non-ambulatory (6.5%)Two ambulatory (6.5%)

**Notes:**

CP, cerebral palsy; GMFCS-E&R, The Gross Motor Function Classification System—Expanded & Revised.

1 Spinal muscular atrophy type 3, Rett syndrome, acquired brain damage, bronchopulmonary dysplasia, microcephaly, and congenital muscular dystrophy.

2 Two acquired brain damage; one muscular atrophy type 3; one Rett syndrome.

A total of 26 patients used the Innowalk size small and the remaining five used size medium. Location of usage of the dynamic standing device was at home or in day care (94%), rarely in hospital, as can be seen in Table 3. Patients practiced dynamic standing for at least 4 weeks (15.7 (4–52) weeks) for 30–60 min (38 (30–43)/session with the frequency from 2 to 7.5 (5 (3–5)) times a week. There was an average of 9.5 ± 3 cumulative hours per month of usage for the individual subjects with a tendency to longer usage of 12 ± 4 h per month after three months (*p* = 0.09).

**Table 3:** Location of the Innowalk usage (*n* = 31).

Place	Frequencies
Home	14 (45.2%)
Day care^1^	15 (48.4%)
Hospital	2 (6.5%)

**Note:**

1 Kindergarten or school in therapeutic institutions.

#### Clinical assessment and questionnaires

Data were collected by paediatricians, physiotherapists, or occupational therapists using standardised questionnaires, at time points before, during, and after usage of the Innowalk. The outcome measures of this pooled study were recorded using the Modified Ashworth Scale (Bohannon & Smith, 1987), the Modified Tardieu Scale (Schädler et al., 2012) or the Duncan–Ely test (Lee et al., 2015) for measurement of spasticity; PROM according to CPOP Manual (2015) for measurement of joint mobility; the GMFM-66 for the gross motor function; GMFCS-E&R (Russel et al., 2002) for gross motor function classification; measurement of circumference of calf and upper leg for measurement of muscle mass; Timed-up and go (Dobson, 2015) fitness, the Saltin-Grimby Physical Activity Level Scale (Grimby et al., 2015) or the International Physical Activity Questionnaire (Saebu & Sorensen, 2011) for estimation of the level of physical activity; the Canadian Occupational Performance Measure (COPM) (Cusick, Lannin & Lowe, 2007) for measurement of performance and satisfaction in self-care, productivity and leisure from the patient’s perspective. The GAS (Kiresuk & Sherman, 1968) was used for scoring the extent to which patients’ individual goals were achieved in the course of intervention, as well as Innowalk standardised protocol (Table 4). Acceptability of the device was recorded by the users’ and care-givers’ subjective impressions, but was not presented here due to small sample size as well as two very different methodological approaches (Tornes et al., 2009; Moen, 2016; Lauruschkus et al., 2017).

**Table 4:** Methodology in the studies.

Author	Questionnaires and methodology
Käferle (2014)	PROM according to CPOP; GMFCS-E&R; Modified Tardieu Scale
Hansen (2014)	GMFM-66; GMFM-88; GMFCS-E&R; Modified Ashworth Scale; PROM according to CPOP
Tornes et al. (2009)	Innowalk standardised protocol (time frame; goals; joint/spasticity measurements; muscle mass; daily documentation on: pain, circulation, gastrointestinal function, sleep patterns)
Moen (2016)	GMFM; GMFCS-E&R; Innowalk standardised protocol
Tollefsen (2015)	Description of video documentation at the baseline and after four weeks of Innowalk use (walking with and without walker and managing to change from sitting to standing, while timing).
Tornes et al. (2012)	GMFM-66; GMFCS-E&R; Modified Ashworth Scale, PROM according to CPOP, circumference of calf and upper leg, questionnaire for parents: use of Innowalk (date, duration), pain, motivation for being in the Innowalk, sleep, gastrointestinal function.
Berner & Ahlborg (2017)	TUG (Timed Up and Go), Saltin-Grimby Physical Activity Level Scale, PROM according to CPOP, Modified Ashworth Scale
Lauruschkus et al. (2017)	MACS; CFCS; GMFCS-E&R; COPM; GAS; GMFM-66; IPAQ; diaries for documenting frequency and length of use
Thon (2012)	GMFM; GMFCS-E&R; Ashworth scale; Rectus femoris lengthening (Duncan Ealy test); circumference of calf and upper leg, gastrointestinal function, description of video film of walking ability with walker at the baseline and after the trial (evaluated by two therapist not knowing the child).

#### Clinical outcome

In five studies including 16 out of 31 patients, individual goals for treatment outcome had been prospectively defined with up to six different individual goals for either joint or muscle function, cardiovascular or intestinal function or overall improvement. These were in detail: improvement of mobility (*n* = 4), muscle strength (*n* = 5), joint mobility (*n* = 3), general physical activity (*n* = 4), walking or weight-bearing transfer (*n* = 4), control/strength of the trunk or head (*n* = 4), COPM (*n* = 5), sleep (*n* = 4), cardiovascular function (*n* = 8), or bowel function (*n* = 15), prevention of deformities/contractures (*n* = 4), or reduction of spasticity (*n* = 1) or frost bites (*n* = 1) or improvement of ‘vital function’ (*n* = 5).

As seen in Table 5, 94% of patients’ clinical outcomes improved. In a relatively short FU period (see above) improvements had been measured for walking or weight-bearing transfer (*n* = 13), control/strength of the trunk or head (*n* = 6), joint mobility (*n* = 14), sleep (*n* = 4 out of 6/67%), or muscle strength (*n* = 17), ‘vital functions’ (*n* = 16), bowel function (*n* = 10), attention/orientation (*n* = 2). The same outcomes were evaluated with similar results across the studies with similar methods of assessment.

**Table 5:** Clinical outcome with the dynamic standing device.

Patient	No. of goals set	No. of goals reached/goals set	No. of improvements/not previously set	Improvement reached but not previously set in studies with prespecified goals	Improvement reached but not previously set in studies without prespecified goals
1 (Moen, 2016)	2	1	3	Muscle strength improved; botulinum toxin dose decreased; no frost bite	
2 (Moen, 2016)	2	1	1	Gastrointestinal function (no need for obstipation medication any more)	
3 (Moen, 2016)	6	0	0		
4 (Moen, 2016)	3	2	0		
5 (Moen, 2016)	5	3	3	Lost weight, improved circulation, and general wellbeing	
6 (Tornes et al., 2012)	6	6	1	Achieved walking with support	
7 (Tornes et al., 2012)	3	3	1	Walking improvement; Improvement of right hip joint (from Reimer’s index 48–39%)—surgery prevented	
8 (Tornes et al., 2012)	3	2	1	Improved mucus mobilizing effect	
9 (Tornes et al., 2012)	3	3	2	Walking improvement + joint mobility	
10 (Berner & Ahlborg, 2017)	1	1	1	Vital functions	
11 (Berner & Ahlborg, 2017)	2	2	1	Walking improvement	
12 (Lauruschkus et al., 2017)	4		1	Vital functions	
13 (Lauruschkus et al., 2017)	4		1	Vital functions	
14 (Lauruschkus et al., 2017)	4		1	Vital functions	
15 (Lauruschkus et al., 2017)	4		1	Vital functions	
16 (Lauruschkus et al., 2017)	4		1	Vital functions	
17 (Tornes et al., 2009)	n. s.	n. s.	n. s.	n. s.	Vital functions (warmer feet) + walking improvement
18 (Käferle, 2014)	n. s.	n. s.	n. s.	n. s.	The breast-belt removed (muscle strength improved); laxative no longer needed
19 (Käferle, 2014)	n. s.	n. s.	n. s.	n. s.	No pain while standing in the standing orthosis; muscle strength increased; regular bowel movement
20 (Käferle, 2014)	n. s.	n. s.	n. s.	n. s.	Increased attention span; head control improved
21 (Tornes et al., 2009)	n. s.	n. s.	n. s.	n. s.	Planned botulinum toxin treatment prevented
22 (Tornes et al., 2009)	n. s.	n. s.	n. s.	n. s.	Circulation improvement
23 (Tornes et al., 2009)	n. s.	n. s.	n. s.	n. s.	Gastrointestinal function improved; stomach pain disappeared; improved muscle strength
24 (Tornes et al., 2009)	n. s.	n. s.	n. s.	n. s.	Muscle strength and walking ability improved
25 (Thon, 2012)	n. s.	n. s.	n. s.	n. s.	Laxative no longer needed; stomach pain disappeared; walking function improved
26 (Käferle, 2014)	n. s.	n. s.	n. s.	n. s.	Spasticity decreased; muscle strength improved
27 (Hansen, 2014)	n. s.	n. s.	n. s.	n. s.	Improved joint mobility
28 (Hansen, 2014)	n. s.	n. s.	n. s.	n. s.	Improved joint mobility
29 (Käferle, 2014)	n. s.	n. s.	n. s.	n. s.	Improved joint mobility
30 (Käferle, 2014)	n. s.	n. s.	n. s.	n. s.	Improved joint mobility
31 (Käferle, 2014)	n. s.	n. s.	n. s.	n. s.	n. s.

**Note:**

n.s., not specified.

#### Passive range of motion of lower extremities

**Figure 3:**
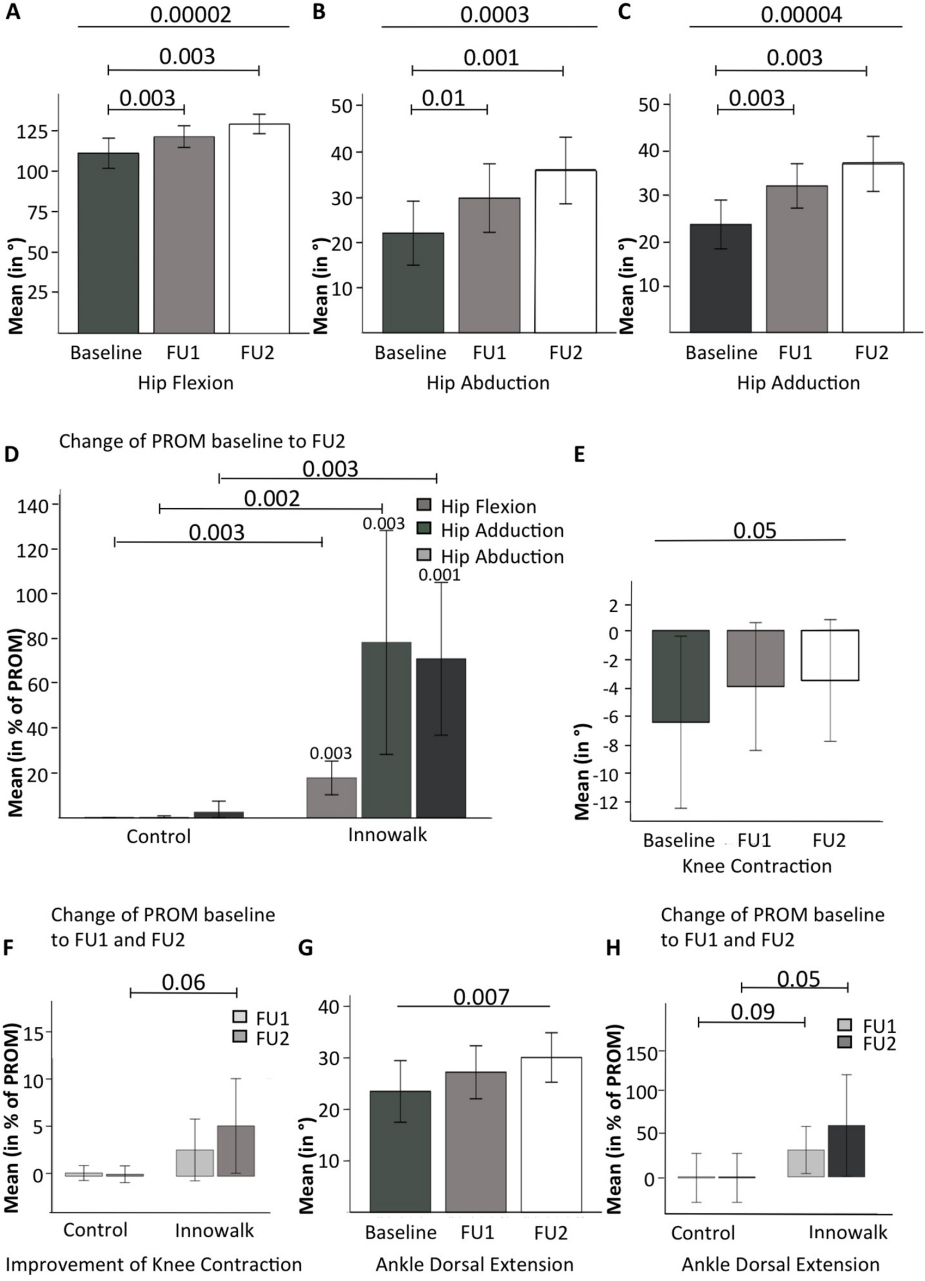
Changes in PROM of the lower extremities with the dynamic standing device. Changes in PROM of the hip joint. (A) flexion, (B) abduction, (C) adduction, and (D) changes in hip PROM with the Innowalk in comparison to control. Improvement of (E) knee contraction, (F) knee contraction, with the Innowalk in comparison to control, (G) ankle dorsal extension, and (H) ankle dorsal extension with the Innowalk in comparison to control. In (D): *p*-values above histograms refer to intra-individual comparisons.

In two studies including a total of nine patients with CP (Hansen, 2014; Moen, 2016), improvement of PROM of the lower extremities had been defined as a prospective goal. As seen from the demographic and clinical data of this subgroup (see Table 6), this subgroup strongly resembles the entire cohort. As shown in Table 7 and Figs. 3A–3C, PROM of the hip significantly (*p* < 0.005 for multiple comparisons) increased after 1 month (FU 1) and 2–5 months (FU 2). The results from the individual studies can be deducted from Table S2. Through direct movement of the Innowalk in this axis, the PROM of hip extension (*p* = 0.06,) and flexion (*p* < 0.0001) were restored. Furthermore, abduction (*p* = 0.0003) and adduction (*p* < 0.0001) as well as internal rotation (*p* < 0.0001) were significantly improved after usage of the Innowalk to a near normal PROM. In contrast (each *p* < 0.005), as demonstrated in Fig. 3D, these changes were not seen in a parallel patient control group with continuous state-of-the-art therapy excluding Innowalk usage. In this relatively small patient group, alleviations of PROM of knee contractions were not significant in comparison to controls (*p* = 0.06, see Table 7; Figs. 3E and 3F) as well as in pes equinus with improvement of dorsal extension of the ankle (*p* < 0.01 for trend) after 2–5 months, as demonstrated in Table 7 and Figs. 3G and 3H, in patients using the Innowalk.

**Table 6:** Baseline characteristics and demographic variables of subgroup with measurements of PROM of lower extremities.

Characteristics	Complete subgroup*	Innowalk**	Control***
	Frequencies or median [IQR]		
Age	7.5 [7–9]	7 [6.5–8.5]	8.5 [7–10]
Age group 1: 2–11 years	13 (100%)	9 (100%)	4 (100%)
Male gender	8 (61.5%)	5 (55.6%)	3 (75%)
Indication			
CP	13	9	4
With spasticity	12	9	3
With spasticity and paresis	1		1
GMFCS-E&R classification			
Level III	1 (7.7%)	1 (11.1%)	
Level IV	3 (23.1%)	2 (22.2%)	1 (25%)
Level V	9 (69.2%)	6 (66.7%)	3 (75%)

**Notes:**

CP, cerebral palsy; GMFCS-E&R, The Gross Motor Function Classification System—Expanded & Revised; *n*, number of patients.

* *n* = 13.

** *n* = 9.

*** *n* = 4.

**Table 7:** Changes in passive range of motion of lower extremities with the dynamic standing device.

	Baseline	FU1	FU2	Significance *p*-values for multiple comparison *n* = 14
Hip extension	2.3 ± 4.5	1.4 ± 3.6	2.2 ± 4.3	n. s.
	0 [0–2.5]	0	0 [0–2.5]	
	n* = 18	*n* = 14	*n* = 18	
Hip neutral extension flexion	−8.21 ± 11.4	−2.6	−2.5	0.06
	0 [−16.3–0]	0 [−6.3–0]	0 [−5–0]	
	*n* = 14	*n* = 14	*n* = 14	
Hip flexion	110.7 ± 16	120.9 ± 14.7	128.7 ± 10.3	0.00002
	115 [93.8–122.5]	126 [110–130]	130 [121.5–140]	
	*n* = 14	*n* = 14	*n* = 14	
Hip abduction	24.2 ± 11.6	29.6 ± 13.1	35.7 ± 11.3	0.0003
	22.5 [11.5–35]	27.5 [20–45]	40 [23.8–36.2]	
	*n* = 18	*n* = 14	*n* = 18	
Hip neutral abduction adduction	0	0	0	
	0	0	0	
	*n* = 14	*n* = 14	*n* = 14	
Hip adduction	23.8 ± 9.3	32.3 ± 8.5	37.1 ± 10.5	0.00004
	20 [17.2–30]	35 [23.8–36.2]	37.5 [28.8–46.2]	
	*n* = 14	*n* = 14	*n* = 14	
Hip external rotation	56.6 ± 31.3	68.8 ± 29	62.8 ± 24.7	n. s.
	47.5 [38.6–80]	70 [40–100]	50 [40–80]	
	*n* = 18	*n* = 14	*n* = 18	
Hip neutral external rotation internal rotation	0	0	0	
	0	0	0	
	*n* = 14	*n* = 14	*n* = 14	
Hip internal rotation	35.9 ± 14.9	43.2 ± 14.5	52.2 ± 19.6	0.00004
	31.8 [23.8–46.2]	40 [30–60]	47.5 [38.7–62.5]	
	*n* = 18	*n* = 14	*n* = 18	
Knee flexion	141.3 ± 46.6	151.4 ± 25.2	155.7 ± 23.8	0.001
	156.5 [143.7–165]	165 [148.7–165]	165 [150–170]	
	*n* = 14	*n* = 14	*n* = 14	
Knee neutral flexion extension	−6.4 ± 10.4	−3.9 ± 7.8	−3.5 ± 7.4	0.05
	−2.5 [−11.2–165]	0 [−6.2–0]	0 [−2.7–0]	
	*n* = 14	*n* = 14	*n* = 14	
Knee extension	1.8 ± 4.6	2.4 ± 5.0	1.8 ± 4.6	n. s.
	0	0 [0–2.2]	0	
	*n* = 14	*n* = 14	*n* = 14	
Ankle dorsal extension	23.4 ± 10.4	27.1 ± 8.9	30 ± 8.3	0.007
	25 [20–31.25]	30 [23.7–30]	30 [25–31.2]	
	*n* = 14	*n* = 14	*n* = 14	

**Note:**

* *n*, number of joints.

#### Safety and acceptability

From all available data, including those with missing key variables, a total of four patients have to be classified either as drop-outs (*n* = 2) or lost-to-follow-up (*n* = 2). Due to the nature of the study, no reports are available on these patients. For the remaining 31 patients of whom quality of data was sufficient to be included in this meta-analysis no adverse events or unacceptable experiences were reported. Adverse events and unacceptable experience with respect to this medical device and with respect to both, paediatric and adult population are: risk for hand injury at the mechanical upright function underneath the seat and column, risk for hand injury at the tilt function of the front frame, redness of the skin in case guide strap is tied to tight to the leg steels, possible redness of the skin and possible allergic reactions where Innowalk comes in contact with the user. The risk of strangulation or other damages could be caused by cables, belts, straps, wires. The risk of foot injury exists in case Innowalk is not used with shoes that fits the Innowalk.

#### Risk of bias assessment

**Figure 4:**
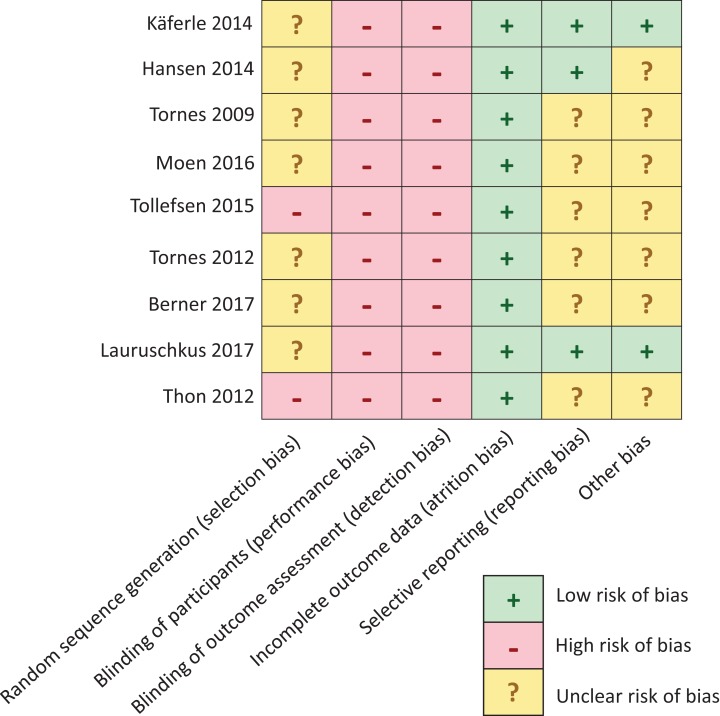
Risk of bias assessment. Quality assessment in this meta-analysis demonstrated a moderate risk of bias in five studies (Käferle, 2014; Hansen, 2014; Tollefsen, 2015; Lauruschkus, Nordmark & Hallstrom, 2017; Thon, 2012) and unclear risk of bias in the remaining four studies (Tornes et al., 2009, 2012; Moen, 2016; Berner & Ahlborg, 2017).

The risk of bias assessment was performed separately for each study. After the first round of iteration, level of agreement was 93% in rating risk of bias. After an additional round of iteration, for the three divergent cases, agreement was readily found. There were no discrepancies that required resolution by a third independent reviewer. Figure 4 provides an overview of biases per domain per study. Five out of nine studies were found to be at moderate risk of bias (Käferle, 2014; Hansen, 2014; Thon, 2012; Tollefsen, 2015; Lauruschkus et al., 2017), while four were found to be at unclear risk of bias (Tornes et al., 2009; Moen, 2016; Berner & Ahlborg, 2017; Tornes et al., 2012). This clearly was because of the small numbers of subjects and the nature of the exploratory case studies. Overall, the strength of evidence for the different reports ranges between medium evidence levels 3 (*n* = 3) (Käferle, 2014; Hansen, 2014; Lauruschkus et al., 2017) to level 5 (*n* = 6) (Thon, 2012; Tollefsen, 2015; Tornes et al., 2009, 2012; Moen, 2016; Berner & Ahlborg, 2017) according to Howick et al. (2011).

## Discussion

The salient finding of this pooled analysis is that regular usage of the Innowalk assistive device in addition to state-of-the-art therapy in this population with a predominantly paediatric population (90%) significantly improved PROM of all joints of the lower extremities in axis. What’s more, additional significant increase in PROM had been gained in the hip for axes not directly affected by the device. Furthermore, for predefined goals in daily living such as improved weight-bearing transfer or walking, general muscle strength, reduction in need for medication or even prevention of planned surgery beyond the effect of standard therapy. This shows the complexity of benefits obtained with this device.

To obtain these data, relevant sites and the internet in general had been searched for groups that had presented data or were conducting ongoing studies with dynamic standing. Generally, two devices were found. Only for one of them—the Innowalk—was data from studies available. Furthermore, the Innowalk manufacturer provided contacts for additional groups of whom they knew that studies were carried out. All but one groups were willing to share their data, whereas one group had key variables missing. Two groups with three ongoing trials obviously could not provide data. This meta-analysis thus summarises all available data world-wide on treatment with this device gathered by scientific groups. The international multicentre approach of this data collection ensures the ascertainment of a sufficient number of cases with a broad spectrum of neuromuscular and functional disorders having been treated. The groups whose data we analysed used similar standardised validated questionnaires and were guided by similar cultural and only minimally diverging national guidelines. Thus, patterns in frequency and intensity of usage, indications, severity of disability deemed elective for usage as well as goals set were similar. Furthermore, these are in line with data from a comparable cohort from Germany derived from real-life prescriptions (manuscript was made available for reviewers).

All of the authors have presented their case series, but none of them has so far performed a sufficiently powered analysis. We tried to overcome these limitations by pooling crude data from distinct yet similar studies involving a generally homogeneous population.

Our results show the diverse improvements related to passive motion of the joints, muscular tone and general well-being associated with physical exercise, for the first time providing evidence in support for beneficial effects of usage of the Innowalk. PROM of hips, knees, and ankles in axis is increased due to direct motion by the device, whereas increases in hip abduction and hip adduction are related to indirect effects, most likely due to reduction of muscle tone, effects on imbalance of antagonising muscle groups. It is tempting to speculate that central nervous stimulation of near-to-physiological leg movements might have a role to this effect. Evidence exists from other devices for this patient group that gravity force (Murri, 1997) and furthermore, weight-bearing exercise (Macdonald et al., 2007, 2008; MacKelvie et al., 2004; Petit et al., 2002; Weeks, Young & Beck, 2008), have positive effects on bone mineral density (Duncan et al., 2002), migration angle of the hip reducing the process of consequent hip luxation and consequent surgical intervention (Scrutton, Baird & Smeeton, 2001). Clearly, increasing muscle strength, or reducing muscle tone will not only improve the described long-term goals, but help reduce the need for medication such as botulinum toxin, as presented here. Improved hip adduction and abduction directly relate to weight-bearing transfer with consequent amelioration of general well-being of patients and caregivers. Similarly, optimised dorsal extension, as shown in our data with the Innowalk, is a prerequisite for firm standing for transfer or perhaps even walking.

Passive motion alone to overcome contractures and to lengthen musculature, increase blood flow and move joints in the physiological range might have first beneficial effects (Goodwin et al., 2017). In addition to the results shown for joints and muscle strength, the Innowalk might improve gastrointestinal function that goes beyond standing devices due to a stimulation of the voluntary trunk musculature. This relates to improvements of mood, ‘vital functions’, sleep, and all relevant parameters to consequently significantly enhance quality of life of patients and caregivers. Reduction of frost bites point towards improved neurovascular regulation, a phenomenon so far unreported. The high rate of acceptability reported here, relates to the fact that data only exists for people who have used the device after an aptitude test and liked the device. Out of all patients in need of assistive standing frames, dislike of using one has been documented for 15–20% of that population (Goodwin et al., 2017). It will be an aspect of future research to confirm the therapeutics experience that dynamic standing is more attractive than mere static standing and identify responders to this therapy.

Cumulatively, the Innowalk is a promising tool with high potential to further improve current evidence-based treatment for patients with severe PD. It is not only for joint mobility and preserving/animating to gait-like movements, but also to significantly enhance relevant aspects related to quality of life. This analysis has limitations inherent to the pooling of data. Data were collected from different cohorts with small numbers from exploratory studies with different standards of acquiring data, quality of documentation, usage of validated questionnaires, that is, for quality of life, and planning of set-up and conduct. Some of the studies had no prespecified goals and thus cannot be considered as prospective studies in strict terms. Patients with flaccid paralyses due to muscular spina bifida, muscular atrophy, or dystrophy have different requirements from patients with CP. Whether these different patient groups might both be suitable for treatment with the Innowalk, and most likely with divergent indications, will have to be considered in future studies. The qualitative components of the reported studies at the pre-trial stage serves the purposes of developing clinically relevant hypotheses, most useful outcome measures and measuring ranges for evaluating this complex intervention method for a heterogeneous study population. These studies gather and present the best evidence available for the effectiveness of dynamic standing and identify the gaps in evidence.

A future prospective randomised and controlled trial with estimated 30 patients according to our power and sample size calculation should be conducted in an international multicentre setting as an add-on to guideline-conform multidisciplinary treatment. A major challenge will be the need to use individually set endpoints measured with agreed upon standardised and validated questionnaires and protocols. The results presented here give an estimate of duration and FU intervals to assess clinically relevant changes. Clearly, a consecutive long-term FU period is necessary to evaluate long-term benefits, for example, bone mineral density, or prevention of hip dysplasia.

## Conclusions

With high similarities of study outcomes in these albeit small studies and bearing the limitations of this analysis in mind, we believe that these data are suitable to form the basis for the urgently warranted randomised, controlled, multicentre trial. Aim of this trial will be to challenge the hypothesis of a standardised usage of the Innowalk for dynamic standing in addition to standard state-of-the-art treatment physiotherapy.

## Supplemental Information

10.7717/peerj.7098/supp-1 Supplemental Information 1 PRISMA checklist. The checklist items pertain to the content of a systematic review and meta-analysis, which include the title, abstract, methods, results and discussion.

10.7717/peerj.7098/supp-2 Supplemental Information 2 PRISMA flow diagram. This figure maps out the number of patients identified, included and excluded, and the reasons for exclusions.

10.7717/peerj.7098/supp-3 Supplemental Information 3 Rationale for conducting the meta-analysis. The reason for this review has been to efficiently integrate existing information and provide quality data for further decision making.

10.7717/peerj.7098/supp-4 Supplemental Information 4 Dataset International Study. Raw data that was used for this meta-analysis.

10.7717/peerj.7098/supp-5 Supplemental Information 5 Cochrane risk of bias tool. Plus (+) represents low risk of bias; minus (−) represents high risk of bias; question mark (?) represents unclear risk of bias.

10.7717/peerj.7098/supp-6 Supplemental Information 6 Results of individual studies in changes in PROM of lower extremities with the dynamic standing device. FU1 represents follow up visit after 1 month. FU2 represents follow up visit in the period from 2-5 months. *n* represents number of joints. In the study of Hansen, there were no control patients (n.d. = not defined).

## Additional Information and Declarations

### Data Availability

The following information was supplied regarding data availability: The raw data is available in Dataset S1.
