# Supplementary figures and images for: Effect of assisted walking-movement in patients with genetic and acquired neuromuscular disorders with the motorised Innowalk device: an international case study meta-analysis
Source: PeerJ. 2019 Jun 18;7:e7098. doi: 10.7717/peerj.7098 (PMC6587941; doi:10.7717/peerj.7098)

Figure 1

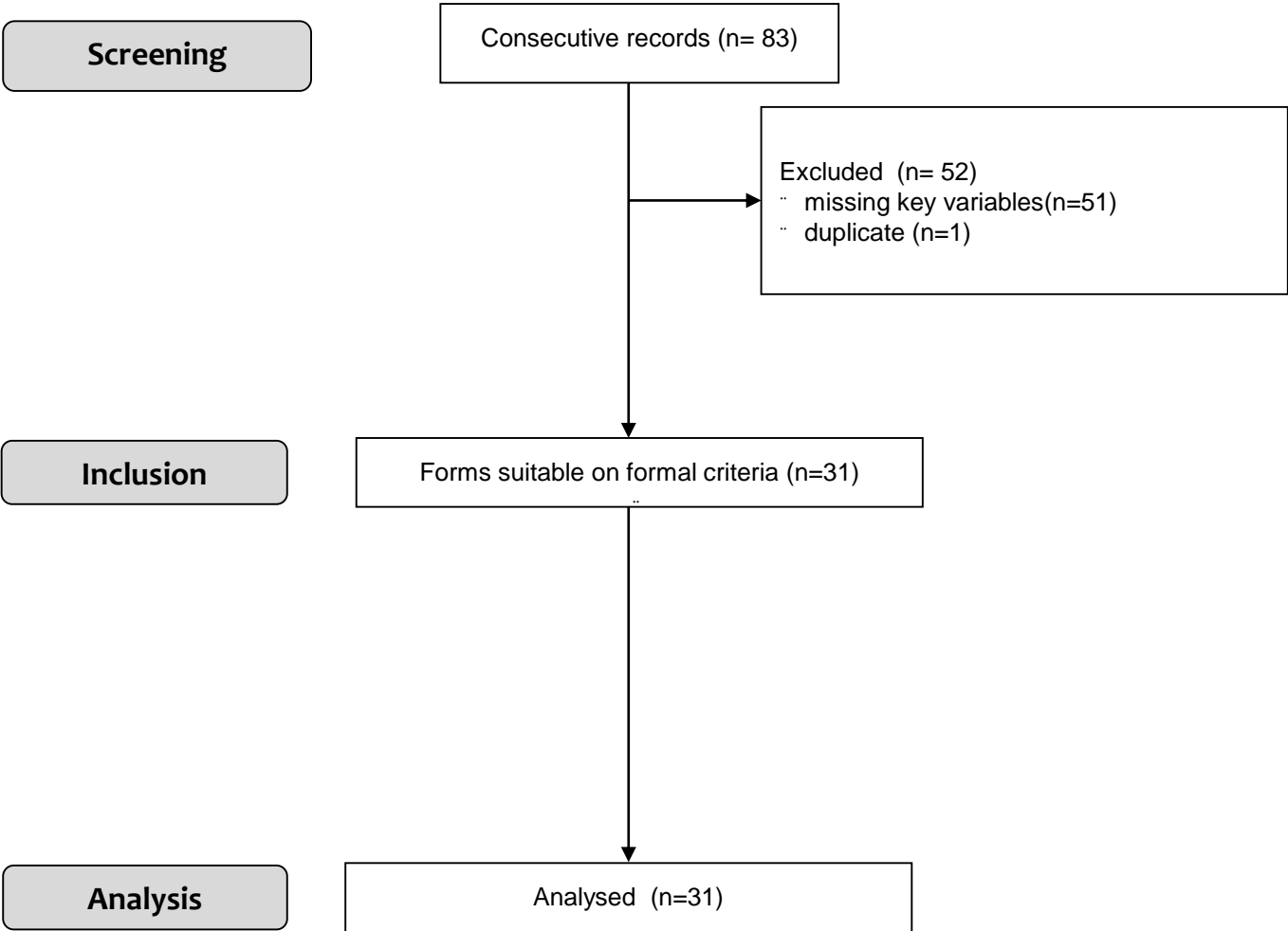

Supplement: Supplemental Information 2 — This figure maps out the number of patients identified, included and excluded, and the reasons for exclusions. [file peerj-07-7098-s002.pdf]
