# Supplementary material for: Effect of assisted walking-movement in patients with genetic and acquired neuromuscular disorders with the motorised Innowalk device: an international case study meta-analysis
Source: PeerJ. 2019 Jun 18;7:e7098. doi: 10.7717/peerj.7098 (PMC6587941; doi:10.7717/peerj.7098)
Supplement: Supplemental Information 3 — The reason for this review has been to efficiently integrate existing information and provide quality data for further decision making. [file peerj-07-7098-s003.docx]

To the

Editor of PeerJ



7. Dezember 2018 07.12.2018

**1. The rationale for conducting the meta-analysis**

The reason for this review has been to efficiently integrate existing information and provide quality data for further decision making. We have collected data from 9 minor studies and tried to establish whether scientific findings are consistent and can be generalised across populations, settings, and treatment variations. Our goal was to increase power and precision of estimates of the Innowalk effects. Finally, we tried to limit bias and improve reliability and accuracy of conclusions.

**2. The contribution that the meta-analysis makes to knowledge in light of previously published related reports, including other meta-analysis and systematic reviews**

This will be the first publication of this type looking at the effects of the assisted walking device in patients with neuromuscular diseases.

**MEDIACC GmbH**

**Medico-academic Consultings**

**Medizinisch-akademische Forschungsberatung**

**Geschäftsführerin: PD Dr. Caroline Schmidt-Lucke**

Telefon +49-(0)30-521 044 80

Telefax +49-(0)30-521 044 83 E-Mail Caroline.Schmidt-Lucke@mediacc.de

Internet www.mediacc.de

MEDIACC GmbH ⏐ Sigmaringer Str. 1 ⏐ 10713 Berlin
