# Supplementary material for: Effect of assisted walking-movement in patients with genetic and acquired neuromuscular disorders with the motorised Innowalk device: an international case study meta-analysis
Source: PeerJ. 2019 Jun 18;7:e7098. doi: 10.7717/peerj.7098 (PMC6587941; doi:10.7717/peerj.7098)
Supplement: Supplemental Information 5 — Plus (+) represents low risk of bias; minus (−) represents high risk of bias; question mark (?) represents unclear risk of bias. [file peerj-07-7098-s005.docx]

# Table S1: Cochrane risk of bias tool. Plus (+) represents low risk of bias; minus (-) represents high risk of bias; question mark (?) represents unclear risk of bias.

| **Domain of bias** | **Qualification** | **Criteria for assigning risk of bias** |
| --- | --- | --- |
| Selection bias |  | Sequence generation |
|  | + | Computer based random number generators, a table with random numbers or similar methods |
|  | - | Quasi randomization procedures e.g. allocation based on date of birth or on day of the week |
|  | ? | None described sequence generation |
| Performance bias |  | Blinding of participants and personnel |
|  | + | Blinded participants and personnel |
|  | - | Non blinded participants and personnel |
|  | ? | None described or unclear blinding of participants and personnel |
| Detection bias |  | Blinding of outcome assessment |
|  | + | Blinded outcome assessor |
|  | - | Non blinded outcome assessor |
|  | ? | Methods of (blinding) the outcome assessment were not described |
| Attrition bias |  | Incomplete outcome data |
|  | + | Random drop-out of participants was present when ≤ 10% drop-out rate |
|  | - | Selective drop-out of participants was present when > 10% dropped-out |
|  | ? | Unclear drop-out rate |
| Reporting bias |  | Selective reporting |
|  | + | Articles that reported all a priori described outcomes |
|  | - | Articles that did not report all a priori described outcomes |
|  | ? | The protocol was not found |
| Other biases |  |  |
|  | + | No other systematic errors were present |
|  | - | Any other systematic errors that could lead to bias (e.g. baseline differences between groups in GMFCS-ER, or other possibly relevant factors) |
|  | ? | Unclear other systematic errors |
