# Supplementary material for: Effect of assisted walking-movement in patients with genetic and acquired neuromuscular disorders with the motorised Innowalk device: an international case study meta-analysis
Source: PeerJ. 2019 Jun 18;7:e7098. doi: 10.7717/peerj.7098 (PMC6587941; doi:10.7717/peerj.7098)
Supplement: Supplemental Information 6 — FU1 represents follow up visit after 1 month. FU2 represents follow up visit in the period from 2-5 months. n represents number of joints. In the study of Hansen, there were no control patients (n.d. = not defined). [file peerj-07-7098-s006.docx]

# Table S2: Results of individual studies in changes in PROM of lower extremities with the dynamic standing device. FU1 represents follow up visit after 1 month. FU2 represents follow up visit in the period from 2-5 months. n represents number of joints. In the study of Hansen, there were no control patients (n.d. = not defined).

|  | | **Baseline** | | **FU1** | | **FU2** | |
| --- | --- | --- | --- | --- | --- | --- | --- |
|  |  | **Control** | **Innowalk** | **Control** | **Innowalk** | **Control** | **Innowalk** |
| Hip extension | Käferle | 0  0  n=8 | 1.4  0  n=14 | 0  0  n=8 | 1,4  0  n=14 | 0  0  n=8 | 1,4  0  n=14 |
|  | Hansen | n.d. | 5.4  5 [0 - 11.2]  n=4 | n.d. | n.d. | n.d. | 5  5 [0 - 10]  n=4 |
| Hip neutral  extension  flexion | Käferle | - 14.8  -19 [-23.8 - -2.5]  n=8 | -8.21  0 [-16.25 - 0]  n=14 | -14.4  -15 [-23.8 - 2.5]  n=8 | -2,6  0 [-6.3 - 0]  n=14 | -14.4  -15 [-23.8 - 2.5]  n=8 | -2.5  0 [-5 – 0]  n=14 |
|  | Hansen | n.d. | n.d | n.d. | n.d | n.d. | n.d |
| Hip flexion | Käferle | 111.3  110 [95 - 128.8]  n=8 | 110.7  115 [93.8 - 122.5]  n=14 | 109.4  110 [91.3 - 127.5]  n=8 | 120.9  126.5 [110 - 130]  n=14 | 109.4  107.5 [95 - 127.5]  n=8 | 128.7  130 [121.5 – 140]  n=14 |
|  | Hansen | n.d. | n.d. | n.d. | n.d. | n.d. | n.d. |
| Hip abduction | Käferle | 20.5  16 [2.5 - 39]  n=8 | 21.9  20 [10 - 35]  n=14 | 19.6  16 [2.5 - 37]  n=8 | 29.6  27.5 [20 - 45]  n=14 | 19.6  16 [2.5 – 37]  n=8 | 35.7  40 [20 - 45]  n=14 |
|  | Hansen | n.d. | 32.2  32 [30.1 - 34.6]  n=4 | n.d. | n.d. | n.d. | 35  35 [30.5 - 39.5]  n=4 |
| Hip neutral abduction adduction | Käferle | 0  0  n=8 | 0  0  n=14 | 0  0  n=8 | 0  0  n=14 | 0  0  n=8 | 0  0  n=14 |
|  | Hansen | n.d. | n.d. | n.d. | n.d. | n.d. | n.d. |
| Hip adduction | Käferle | 22.5  22.5 [18.5 – 28.7]  n=8 | 23.8  20 [17.3 - 30]  n=14 | 22.87  22.5 [18.5 – 30]  n=8 | 32.3  35 [23.7 – 36.2]  n=14 | 22.87  22.5 [18.5 – 30]  n=8 | 37.1  37.5  [28.8 – 46.2]  n=14 |
|  | Hansen | n.d. | n.d. | n.d. | n.d. | n.d. | n.d. |
| Hip external rotation | Käferle | 49.4  42.5 [25 - 78.8]  n=8 | 60.1  50 [36.3 - 85]  n=14 | 50  45 [25 – 78.8]  n=8 | 68.8  70 [40 - 100]  n=14 | 50  45 [25 – 78.8]  n=8 | 67.14  70  [40 - 85]  n=14 |
|  | Hansen | n.d. | 44.4  44.7 [37.3 - 51.2]  n=4 | n.d. | n.d. | n.d. | 47.5  50  [42.5 - 50]  n=4 |
| Hip neutral external rotation internal rotation | Käferle | 0  0 | 0  0 | 0  0 | 0  0 | 0  0 | 0  0 |
|  | Hansen | n.d. | n.d. | n.d. | n.d. | n.d. | n.d. |
| Hip internal rotation | Käferle | 47.5  47.5 [28.8 – 60  n=8 | 34.8  30 [20 - 43]  n=14 | 47.5  47.5 [28.8 – 60  n=8 | 43.2  40 [30-60]  n=14 | 47.5  47.5 [28.8 – 60  n=8 | 53.6  45 [33.8-73.8]  n=14 |
|  | Hansen | n.d. | 39.7  38.8 [31.4 – 48.8]  n=4 | n.d. | n.d. | n.d. | 47.5  50 [42.5-50]  n=4 |
| Knee flexion | Käferle | 155.4  157.5 [148.5 – 160]  n=8 | 141.3  156.5 [143.8 – 165]  n=14 | 156  157 [150 – 160]  n=8 | 151.4  165 [148.8 – 165]  n=14 | 155.8  157.5 [148.5 – 160]  n=8 | 155.7  165 [150 – 170]  n=14 |
|  | Hansen | n.d. | n.d. | n.d. | n.d. | n.d. | n.d. |
| Knee neutral flexion extension | Käferle | - 22.2  -20 [-44.5 – (- 2.5)]  n=8 | - 6.4  - 2.5 [-11.2 – 0]  n=14 | -23.8  -20 [-48.8 – (- 2.5)]  n=8 | - 3.9  0 [-6.2 – 0]  n=14 | -23.8  -20 [-48.8 – (- 2.5)]  n=8 | - 3.5  0 [-2.8 – 0]  n=14 |
|  | Hansen | n.d. | n.d. | n.d. | n.d. | n.d. | n.d. |
| Knee extension | Käferle | 5  0 [0 – 15]  n=8 | 1.8  0  n=14 | 5  0 [0 – 15]  n=8 | 2.4  0 [0 – 2.5]  n=14 | 5  0 [0 – 15]  n=8 | 1.8  0  n=14 |
|  | Hansen | n.d. | n.d. | n.d. | n.d. | n.d. | n.d. |
| Ankle dorsal extension | Käferle | 20.2  26 [5 – 30]  n=8 | 23.4  25 [20 – 31.2]  n=14 | 19.4  20 [5 – 30]  n=8 | 27.1  30 [23.8 – 30]  n=14 | 19.4  20 [5 – 30]  n=8 | 30  30 [25 – 31.2]  n=14 |
|  | Hansen | n.d. | n.d. | n.d. | n.d. | n.d. | n.d. |
